# Designing iCanFit: A Mobile-Enabled Web Application to Promote Physical Activity for Older Cancer Survivors

**DOI:** 10.2196/resprot.2440

**Published:** 2013-02-14

**Authors:** Yan Hong, Deborah Vollmer Dahlke, Marcia Ory, Angela Hochhalter, Jana Reynolds, Ninfa Pena Purcell, Divya Talwar, Nola Eugene

**Affiliations:** ^1^Department of Health Promotion and Community Health ScienceSchool of Rural Public HealthTexas A&M Health Science CenterCollege Station, TXUnited States; ^2^Texas Life Science FoundationAustin, TXUnited States; ^3^Scott & White HealthcareTemple, TXUnited States; ^4^Texas AgriLife Extension AgencyTexas A&M University SystemCollege Station, TXUnited States

**Keywords:** older cancer survivors, physical activity, survivorship, mHealth, website design, user-computer interface, protocol development, formative research

## Abstract

**Background:**

Most older cancer survivors (OCS) do not engage in regular physical activity (PA) despite well-known health benefits. With the increased use of mobile technologies among older adults, mobile tools may be an effective method to deliver PA promotion programs for OCS.

**Objective:**

To document the process of designing an OCS-friendly mobile-enabled Web application of PA promotion program.

**Methods:**

Mixed methods encompassing group discussions, individual interviews, and brief surveys with community leaders, OCS, cancer care providers, and software professionals were used in this formative research.

**Results:**

The varied stakeholders welcomed the idea of developing an online tool to promote PA in OCS. Our formative research revealed several major barriers to regular PA including limited access to senior-friendly PA resources, lack of motivation and social support, and insufficient knowledge and skills on building safe and appropriate workout plans. This feedback was incorporated into the development of iCanFit, a mobile-enabled Web application, designed specifically for OCS. The iCanFit online tools allow users to locate PA resources, set and track goals for PA, network with peer OCS in a secure online space, and receive practical and evidence-informed healthy tips.

**Conclusions:**

Our mixed-method formative research led to the design of iCanFit protocol to promote PA and well-being of OCS. The involvement of stakeholders is critical in the planning and design of the mobile application in order to enhance program relevance, appeal, and match with the needs of target users.

## Introduction

Early detection and improved treatment for cancer has resulted in approximately 13 million survivors in the Unites States today. It has been estimated that these numbers will increase by nearly a third to 18 million by 2022 [1]. Most cancer survivors are 60 years of age or older, and the population of older cancer survivors (OCS) is increasing, resulting in many challenges associated with the continuity of care and resultant quality of life [2,3]. Countering these challenges, numerous studies point to the physiological and psychosocial benefits of physical activity (PA) for cancer survivors [4,5]. These benefits include improved physical endurance, capacity and strength, higher immune function and hemoglobin concentration, decreased risk of cancer recurrence, and improved quality of life as indicated by decreased fatigue and depression [6-10]. Several guidelines on PA for cancer survivors have been recently issued, recommending cancer survivors to engage in low to moderate impact PA regularly, specifically, at least 30 minutes of exercise a day for 5 days a week [11,12].

Despite the recognized importance of PA for cancer survivors, evidence suggests that the level of adherence to PA guidelines among cancer survivors is very low. Smith and Chagpar reported that only 4.6% of breast cancer survivors followed PA guidelines, compared to 12% of women without breast cancer [13]. The majority of cancer survivors are not participating in the recommended levels of PA, resulting in a greater disease risk and health care costs [14]. In a review of existing programs to promote PA among cancer survivors, Schmitz and colleagues observed that most existing programs are limited to walking only, and that there is a lack of diversity in types of PA for cancer survivors [15]. In addition, most existing studies of interventions to increase PA have relied on individual or small group counseling approaches, typically offered by nurses or health educators. Such face-to-face approaches, although promising, have encountered problems of high personnel cost and limited reach. Thus, a critical need exists for more cost-effective strategies to reach a large number of OCS with effective strategies for promoting PA [12,16].

In the exploration of interventions to promote PA among OCS, mobile technologies are gaining increased attention across all age groups. As of 2012, about 88% of US adults and nearly 70% of people older than 50 accessed the Internet. 85% of US adults owned cell phones, of those, 53% owned smartphones [17]. Approximately 70% of adults over 50 owned a cell phone, and more than 42% owned a laptop or tablet computer [18]. The percentage of older smartphones or mobile computer owners is expected to accelerate in the coming years. Further, recent surveys by National Cancer Institute indicated that more than 60% of cancer survivors access the Internet; and once online, they are more likely to use it for health-related purposes than general users [19, 20]. Among cancer survivors, the Internet has become the second most used resource for finding health-related information (the primary resource was physicians) [21,22]. The ubiquity of Internet access in the US and the rapid penetration of smartphones and tablets suggests the feasibility of using mobile technologies to promote PA among OCS. In a recent review of existing Web and mobile applications for PA promotion, we found that most websites or applications were designed for general and younger populations. Few online PA programs were designed for cancer survivors, not to mention OCS. The purpose of this article was to describe a formative study we conducted to inform the design of a mobile or Web application for the purpose of promoting PA among OCS.

## Methods

### Overview

A multidisciplinary research team from Communities of Texas: Cancer • Activity • Research • Education • Support (CTxCARES) program representing public health, medicine, health education, and technology development worked collaboratively to design the formative research protocol. A mixed-method approach was used to collect data to inform the structure and content of the mobile or Web application. Our team met regularly to discuss the data collected from field, enabling prompt feedback for project development and refinement. Data collection in formative research spanned from November 2011 to September 2012. We held group discussions with community leaders, individual interviews with cancer care providers, and conducted a brief survey and interviews with OCS. Table 1 lists all the data collection methods utilized. The study protocol was approved by the Institutional Review Boards at the Texas A&M University and Scott & White Healthcare.

**Table 1:** Data sources of formative research in the iCanFit program.

Data collection method	Participants	Recruitment venues	Data collection mode
Group discussion	Community leaders (n=20)	Community centers	Face-to-face
Individual interview	Cancer care providers (n=14)	Conferences, clinics	Face-to-face, phone
Individual interview	Older cancer survivors (n=20)	Communities, clinics, cancer-related events	Face-to-face, phone
Questionnaire	Older cancer survivors (n=92)	Communities, cancer-related events	Paper-pencil

### Group Discussions with Community Leaders

The CTxCARES program has collaborated with local cancer communities and senior communities for several years, leading to established relationships. At the planning stage of the project, we communicated about our intention to develop a mobile or Web application for OCS to local senior and cancer community leaders and obtained their support. In our formative research, we had 2 group discussions with local community leaders to get their input on the feasibility of a website to engage OCS and promote their PA. The discussions took place in informal expanded meetings of community organizations in which community leaders and members were present. These discussions were not taped, but detailed notes were taken during and after the meeting.

### Interviews with Cancer Care Providers

We also interviewed practicing cancer care providers who have patients either in active cancer treatment or are cancer survivors. The provider’s interview guide was developed based on the literature review and informal discussions with community leaders. The guide focused on: (1) perceptions on OCS’ barriers to regular PA, (2) current counseling of PA with OCS, (3) suggested contents of a mobile or online program, and (4) strategies to disseminate the program after piloting. The interview guide was piloted with 2 physicians before field use. A total of 14 cancer care providers were recruited during a large state cancer conference, representing surgeons, oncologists, family physicians, internists, health educators, and counselors. One-on-one interviews ranging from 20-45 minutes were conducted in a private space. All conversations were audio-taped and transcribed verbatim.

### Survey with OCS

We conducted a brief survey with OCS who were at least 60 years old, had a cancer diagnoses, and had completed initial treatment. Surveys were distributed at local health fairs and cancer survivors’ events. Participants reported on their Internet and cell phone use, current PA behaviors, access to health information, and attitudes toward mobile-based PA promotion program. It took the OCS approximately 10 minutes to complete the brief paper-pencil survey. A total of 92 OCS (mean age 62) provided oral consent and completed the survey. All survey data were entered into SPSS 16.0 (SPSS Inc, Chicago, IL, USA).

### Interviews with OCS

Our third strategy was to conduct in-depth individual interviews with OCS. We developed an interview guide for OCS based on the brief survey with OCS and the in-depth interviews with cancer care providers. The interview guide consisted of the following domains: (1) current PA including type, intensity, duration, and frequency, (2) barriers to regular PA, (3) preferred PA and facilitators to regular PA, (4) attitudes toward an online or mobile PA promotion program, (5) suggestions on content and features of the mobile program, and (6) suggestions on program implementation and evaluation. A total of 11 OCS in Central Texas were recruited through community outreach strategies. The interviews were conducted face-to-face or over the phone, lasted 25-45 minutes, and were audio-taped. Each participant provided consent and received a $15 gift card.

### Data Analysis and Interpretation

The data collected in the formative research included: (1) notes from the research team’s regular meeting discussions, (2) detailed notes from discussions with community leaders, (3) transcripts from interviews with cancer care providers, (4) survey data from OCS, and (5) transcripts from interviews with OCS. Quantitative data were saved in SPSS and descriptive statistical methods were used to analyze frequency of key variables. Qualitative data were saved in Microsoft Word; thematic content analysis was used to identify the major themes and key exemplary quotes [23]. Findings from each data set were used to inform analysis and interpretation of other data sets. The data analysis was iterative and continued throughout the formative research; the findings were used to inform the design, content, and structure of the iCanFit mobile-enabled Web application.

## Results

### Feasibility of Mobile PA Promotion Program for OCS

The survey and interviews with OCS and discussions with community leaders revealed the current online behaviors of OCS. Most of OCS accessed the Internet, and the Internet was a major source of health information for the participants. For example, more than 80% of OCS in our interviews indicated that they would participate in an online PA promotion program or OCS support program. OCS also suggested that compared to younger people, they had shorter online duration and that they were more likely to visit the same authoritative sites. Less than 10% of OCS accessed the Internet through their cell phones frequently, but many had tablets such as iPads and many accessed the Internet through their mobile devices.

I use Internet many times a day, and I search for health information for myself and my family. I search the Internet using both my desktop and my iPhone…People have misconceptions about seniors using mobile technologies, many of us are computer savvy.

61 year old OCS

Some OCS, especially those over 75 years old, reported less use of Internet themselves, but they relied on their families to access the Internet and obtain health information. More than 80% of the OCS we talked to have easy access to health information and could easily obtain this information from the Internet.

### Barriers to PA Among OCS

The interviews with cancer care providers indicated that most of them were aware of regular PA as part of healthy living for cancer survivors, but very few discussed PA with their survivor patients. Care providers identified the lack of motivation to carry out PA from survivors as the major barrier to regular PA. For example, a physician related, “If they want to do it, they’ll do it. If they don’t, they won’t.” Most cancer care providers listed time constraint as a major barrier to discussing PA with their cancer survivor patients. As one physician indicated, “The conversation doesn’t get that in-depth because we have so many other issues to discuss.”

The interviews with OCS confirmed that most did not participate in regular PA. Four personal and structural level barriers to PA were identified. First, some survivors were not aware of the benefits of PA.

For myself, since I got colon cancer, I became more cautious with what I eat. I see nutrition is more important for me…Physical activity? I do some yard work, and when I shop in Wal-Mart, I walk fast with my shopping cart

60 year old OCS

Second, some understood the importance of regular PA, but lacked motivation to be more physically active.

After all the treatments, I just want to go back to my normal life. I know exercise is good for health, but it is probably the last thing in the mind now. I just want to get back to normal, rather than an enhancement or set up new goals.

67 year old OCS

Third, many OCS had limited access to information on local, senior-friendly PA programs and resources. They expressed frustration in finding senior-friendly PA resources.

There is a senior center here in [omitted], but I’ve been there and looked into their exercise room and it’s about twice the size of my hall bathroom; and it is so confined that I am not inspired to get in there and do anything.

70 year old OCS

Another OCS who had tried some programs said, “It did help a little but I quit going because they did not treat me as an individual but they just do everybody in mass.” In addition, since PA was rarely discussed in their visit with health care providers, OCS did not know how to set up individualized plans that fit their health and needs. Fourth, some OCS did not have enough social support, especially peer support, to engage in regular PA. For instance, some OCS commented, “I am basically lazy…and I need somebody to crack the whip…If I had an exercise partner, I may go more often.” Our brief survey with OCS suggested that only a small number of OCS were part of the online cancer support groups but most were willing to join one.

### Key Functions and Structure of the Online PA Promotion Program

In addition to identifying barriers to regular PA, our participants of community leaders, cancer care providers, and OCS also provided suggestions on how to design the online program to address these barriers. Their input was incorporated into the design of a mobile-enabled Web application called iCanFit. The reason for a Web application instead of a mobile application is to fulfill the goal of making iCanFit accessible through all mobile devices with Internet connections. Most mobile applications are limited to certain devices or platforms. For example, iPhone apps will not work on Android phones. Our mobile-enabled interactive website allows seniors to access the program through their various mobile devices with minimal restrictions.

Table 2 lists input from stakeholders on the barriers and corresponding functions on the Web application to address stakeholder feedback. The interactive iCanFit has the following 6 major functions addressing the needs and barriers to regular PA in OCS:

1. “Locator”, a function that allows easy search for local resources of PA, including parks, facilities, and programs. Users can set their preferences for searching, for example, indoors, free, group activity, etc.

2. “Goals”, a function that allows users to set long- and short-term goals of PA. They can track their PA by entering their weekly PA. The iCanFit system will provide tailored feedback on their progress and suggestions on changing on short-term goals. iCanFit sends weekly reports to the users tracking their goals and providing tailored tips and kudos. Those who met their goals were congratulated by a virtual coach and those who did not meet their goals received motivational messages from the virtual coach.

3. “Community”, a function that offers social support through virtual networking. Users are able to create a profile, post images, and start and follow discussions and to connect with peers users. Users are also able to create a profile, post images, and start and follow discussions and to connect with peers users. Those who exceed the short-term PA goals will also be recognized and congratulated in the online community.

4. “Healthy Tips”, a function that sends regular health tips to users. These consist of tips for seniors to make small changes in daily life and make incremental progress toward an active and healthy living.

5. “Library”, is a function that provides convenient access to authoritative health information. In addition to links to major senior health and cancer survivor organizations, this function also allows keyword search for specific health information.

6. “Support”, is a function where users can seek help and technical support when using iCanFit programs.

**Figure 1:**
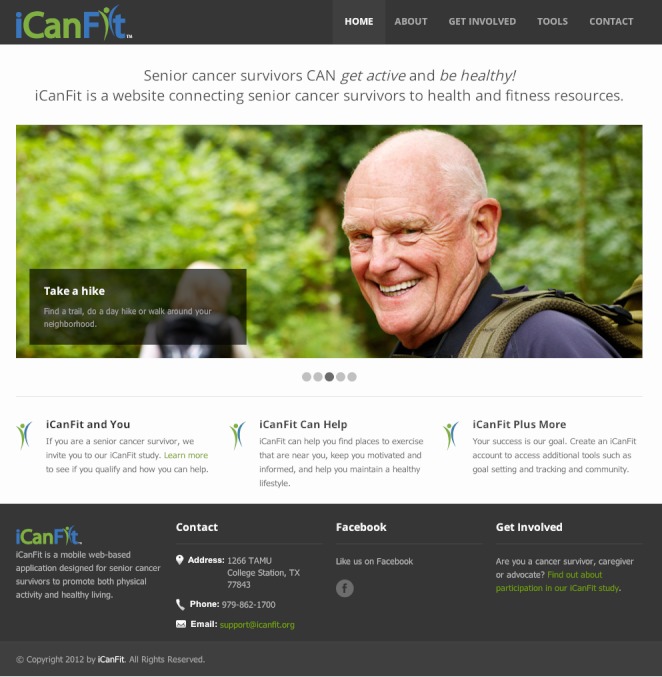
iCanFit webpage interface on a desktop computer.

**Figure 2:**
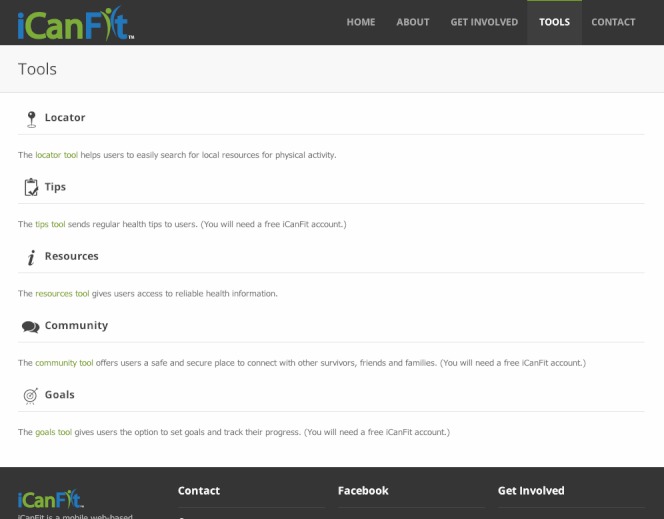
Major functions of iCanFit on desktop interface.

**Figure 3:**
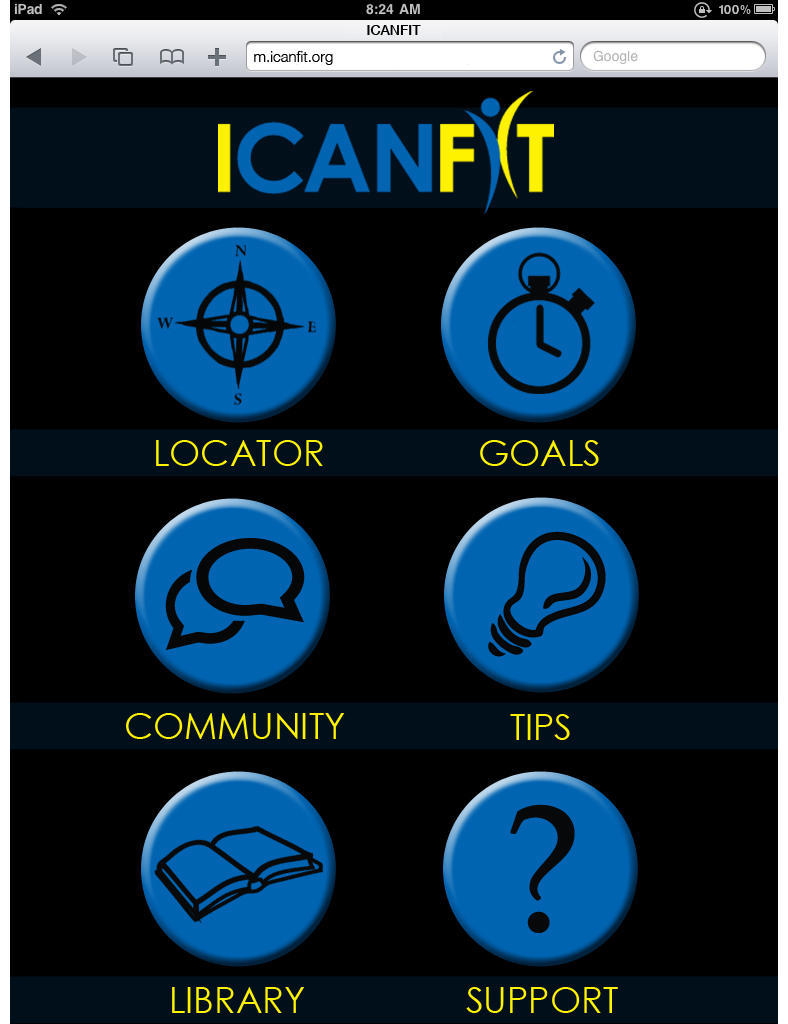
Major functions of iCanFIt on an iPhone interface.

We included some screen shots to help visualize how iCan Fit works. Figure 1 depicts iCanFit homepage on a desktop, Figure 2 displays the key functions of iCanFit on a laptop, and Figure 3 shows iCanFit functions on an iPhone interface. In line with stakeholders’ suggestions and the National Library of Medicine guidelines of “making your website senior friendly” [24], the iCanFit interface is simple with bigger icons, bigger fonts, more space between lines, and clear background in contrast with the main texts. The iCanFit protocol is currently being developed into active Web application.

**Table 2:** Proposed key iCanFit functions based on inputs from stakeholders.

Input	Proposed iCanFit function	Purpose
OCS: Limited safe and senior-friendly physical activity resources. CL: Lack of access to information on community resources and events.	Locator	Allows easy search for local resources of PA, including parks, facilities, and programs Users can set their preferences for searching, for example, indoors, free, group activity etc Suggests a variety of types of PA
CCP: Insufficient time to discuss PA or make specific plans with patients. OCS: Need assistance in setting individualized plans.	Goals	Allows users to set long- and short-term goals of PA. Users can track their PA and iCanFit provides tailored feedback
OCS: OCS need social support, especially peer support to start and maintain regular physical activity. OCP: Social support is an important factor for OCS to stay physically active. CL: Online community can expand OCS social networks, especially for rural residents.	Community (Chatter)	Offers social support with other OCS through virtual networking Users can create personal profiles, post images, start and follow discussions
CCP: OCS lack motivation to stay physically active. OCS: Need constant reminders to continue the momentum. CL: Simple tips can make small changes, and small changes can add up.	Healthy Tips	Sends regular practical, senior-friendly, evidence-based healthy tips specific to OCS
OCS: Many OCS are unaware of necessity and benefits of regular PA. CCP: Do not have enough time to carry out patient education.	Library	Provides convenient access to authoritative health information Provides links to major senior health and cancer survivor organizations

## Discussion

With the population of OCS expanding and few OCS having regular PA, a strong need exists for a cost-effective health promotion program targeting this population. Online access is increasingly common among older adults, and mobile use is also accelerating, thus offering a potential mode of widespread intervention delivery. A combination of Web and mobile technology that incorporates the best principles of chronic care management—including identifying patient preferences, addressing barriers to lifestyle changes, enhancing self-management skills, providing coaching and feedback, and facilitating peer support—offers an excellent opportunity to meet this need [25,26].

Although the number of online health promotion programs is skyrocketing with demonstrated efficacy [27,28], very few programs are available for OCS. This study documents the process necessary to develop a PA promotion program for OCS. It has long been recognized that employing user-centered design and development process is essential for ensuring a quality user experience [29-31]. Our formative research has involved the key stakeholders from very beginning and throughout the phase of protocol development. The resulting iCanFit protocol addresses OCS’ barriers to regular PA. It provides an engaging venue for achieving behavioral change through easy access to community resources, goal setting and reinforcement, and peer support. It is important that these functions are relevant to OCS and presented in a format that appeal to the intended audience.

Several limitations should be noted for this study. First, we had a relatively small sample size in both quantitative and qualitative data collection, which might introduce potential reporting bias. However, our participants were drawn from a diverse background and the data suggested saturation. Second, as our participants were recruited from communities in Texas, the findings may not be generalizable to OCS from other locations. We employed community outreach versus online recruitment because we needed to conduct an in-depth and sometimes repeated interviews with OCS to obtain their feedback. Third, our program only addressed 3 key barriers to regular PA identified by OCS and their care providers. Whether the program met their expectations and could promote regular PA and health outcomes requires a long-term study. We are now developing the iCanFit protocol to the active Web application. Once beta-testing is completed, we will conduct an efficacy trial to assess whether it can promote PA and other health outcomes of OCS.

The design of online health promotion programs, especially behavioral change programs, should reflect collaborative efforts between researchers, computer software designers, and key stakeholders. Through this formative research, we learned the importance of setting a realistic timeline and recruiting participants from diverse backgrounds. We also appreciate the value of a mixed-method study that involves key stakeholders to understand target users’ needs and preferences in order to identify best strategies for program development and evaluation of this new mobile-enabled Web application.

## Abbreviations

CCP: cancer care providers
CL: community leaders
CTxCARES: Communities of Texas: Cancer • Activity • Research • Education • Support
OCS: older cancer survivors
PA: physical activity
